# 641. A Care Continuum for Doxycycline as Post-Exposure Prophylaxis against Bacterial Sexually Transmitted Infections in Black Sexual Minority Men

**DOI:** 10.1093/ofid/ofaf695.205

**Published:** 2026-01-11

**Authors:** Samuel Opara, Sabriya L Linton, McKinsey Bullock, Srija Dutta, Antonio Newman, Marcus O Reed, Daniel I Alohan, Nata M Assad, Leslie M Carson, Kamini Doraivelu, Kyle J Moon, Tsedenia Tewodros, Brian W Weir, Sophia A Hussen

**Affiliations:** Emory University, Atlanta, GA; Bloomberg School of Public Health, Johns Hopkins University, Baltimore, Maryland; Emory University, Atlanta, GA; Emory University, Atlanta, GA; Emory University, Atlanta, GA; Emory University, Atlanta, GA; Emory University, Atlanta, GA; Emory University, Atlanta, GA; Bloomberg School of Public Health, Johns Hopkins University, Baltimore, Maryland; Emory University, Atlanta, GA; Bloomberg School of Public Health, Johns Hopkins University, Baltimore, Maryland; Emory University, Atlanta, GA; Bloomberg School of Public Health, Johns Hopkins University, Baltimore, Maryland; Emory University, Atlanta, GA

## Abstract

**Background:**

Rates of sexually transmitted infections (STIs) have increased in recent years in the US, with racial and sexual minority populations bearing a disproportionate burden of disease. Doxycycline for post-exposure prophylaxis (doxyPEP) has emerged as a novel biomedical preventive strategy for bacterial STIs in men who have sex with men and transgender women. We developed a care continuum for doxyPEP in a cohort of Black sexual minority men (SMM) in Atlanta, Georgia and described the association between recent bacterial STI and each stage of the continuum.

**Figure:**
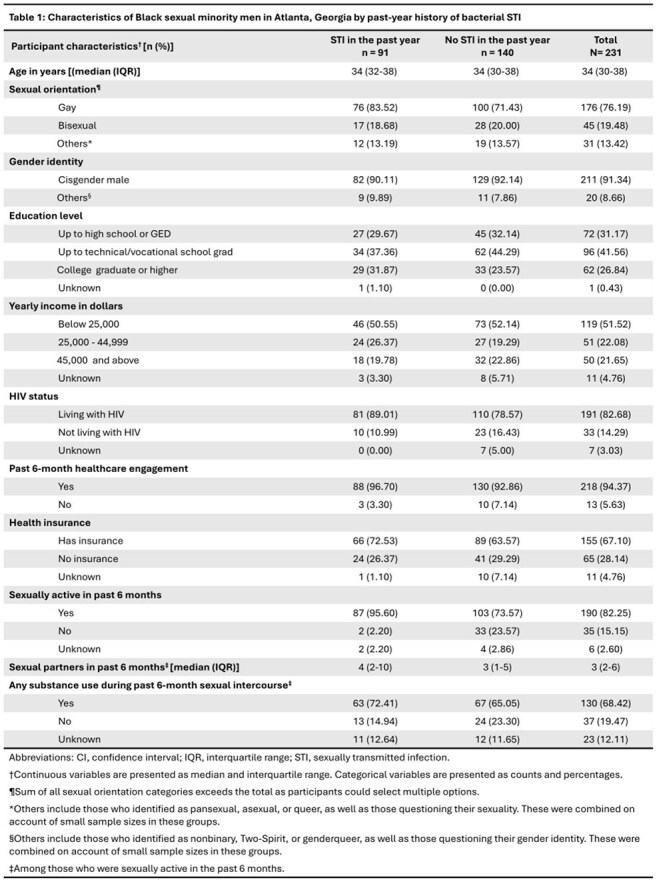

**Figure:**
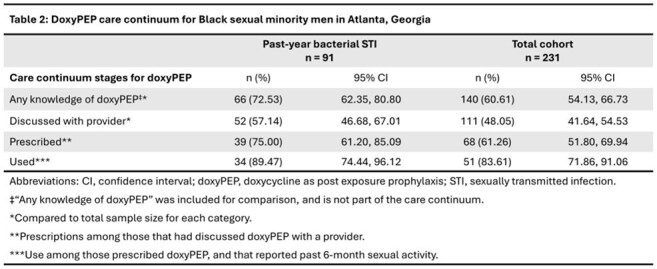

**Methods:**

We analyzed cross-sectional, self-reported data from 231 Black SMM in Atlanta, aged 18-44 years, enrolled in the HISTORY study. We dichotomized participants by past year bacterial STI status and described both groups by sociodemographic, sexual behavior and sexual health characteristics. Based on published guidelines, the stages of our doxyPEP care continuum were 1) discussion with a healthcare provider, 2) prescription, 3) use. We computed the proportion of participants in each stage of our care continuum among the entire cohort, and among the subset that reported any bacterial STI in the past year (gonorrhea, chlamydia, syphilis). Using log-binomial regression for each stage of the continuum, we computed crude prevalence ratios comparing those with recent bacterial STI to those without.

**Figure:**
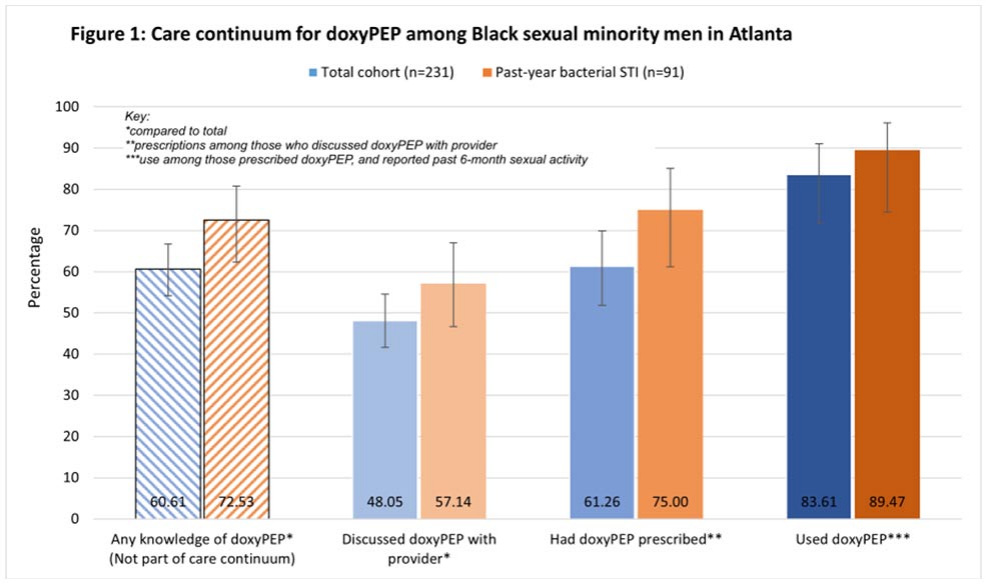

**Figure:**
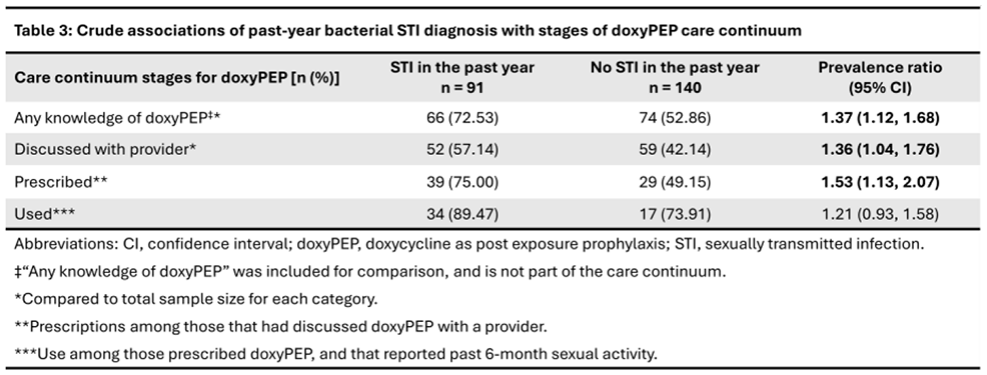

**Results:**

Median age of participants was 34 years (IQR 30-38). Most participants were living with HIV (82.68%). Those who reported past 6-month sexual activity had a median of 3 sexual partners (IQR 2-6; Table 1). Prevalence of recent bacterial STI was 39.39%. Stages of the care continuum are presented in Table 2 and Figure 1. Those with recent bacterial STI were more likely to have 1) discussed doxyPEP with a provider (PR 1.36; 95% CI 1.04, 1.76), and 2) been prescribed doxyPEP (PR 1.53; 95% CI 1.13, 2.07). There was no difference in doxyPEP use between those with recent bacterial STI and those without (PR 1.21; 95% CI 0.93, 1.58).

**Conclusion:**

Our findings suggest high levels of use among Black SMM prescribed doxyPEP, but suboptimal levels of provider-driven conversations about doxyPEP. Future efforts should incorporate doxyPEP counseling into preventive sexual health discussions and increase public awareness of doxyPEP among this high priority group.

**Disclosures:**

All Authors: No reported disclosures

